# Review of neurodevelopmental disorders in patients with HNF1B gene variations

**DOI:** 10.3389/fped.2023.1149875

**Published:** 2023-03-09

**Authors:** Clara Marie Nittel, Frederike Dobelke, Jens König, Martin Konrad, Katja Becker, Inge Kamp-Becker, Stefanie Weber, for the NEOCYST consortium

**Affiliations:** ^1^ Department of Child and Adolescent Psychiatry, Psychosomatics and Psychotherapy, Philipps University, Marburg, Germany; ^2^ Department of General Pediatrics, University Children’s Hospital, Münster, Germany; ^3^ Department of Pediatric and Adolescent Medicine, Philipps University, Marburg, Germany

**Keywords:** renal cyst and diabetes syndrome, HNF1B, neurodevelopmental disorder (NDD), autism spectrum disorder, review

## Abstract

This review investigates the association between neurodevelopmental disorders (NDD) and variations of the gene HNF1B. Heterozygous intragenetic mutations or heterozygous gene deletions (17q12 microdeletion syndrome) of HNF1B are the cause of a multi-system developmental disorder, termed renal cysts and diabetes syndrome (RCAD). Several studies suggest that in general, patients with genetic variation of HNF1B have an elevated risk for additional neurodevelopmental disorders, especially autism spectrum disorder (ASD) but a comprehensive assessment is yet missing. This review provides an overview including all available studies of patients with HNF1B mutation or deletion with comorbid NDD with respect to the prevalence of NDDs and in how they differ between patients with an intragenic mutation or 17q12 microdeletion. A total of 31 studies was identified, comprising 695 patients with variations in HNF1B, (17q12 microdeletion *N *= 416, mutation *N *= 279). Main results include that NDDs are present in both groups (17q12 microdeletion 25.2% vs. mutation 6.8%, respectively) but that patients with 17q12 microdeletions presented more frequently with any NDDs and especially with learning difficulties compared to patients with a mutation of HNF1B. The observed prevalence of NDDs in patients with HNF1B variations seems to be higher than in the general population, but the validity of the estimated prevalence must be deemed insufficient. This review shows that systematical research of NDDs in patients with HNF1B mutations or deletions is lacking. Further studies regarding neuropsychological characteristics of both groups are needed. NDDs might be a concomitant of HFN1B-related disease and should be considered in clinical routine and scientific reports.

## Introduction

Neurodevelopmental disorders (NDDs) are characterized by an impairment in cognition, communication, behavior and/or motor skills originating from abnormal brain development (1). According to the *Diagnostic and Statistical Manual of Mental Disorders*, Fifth Edition (2), NDDs include, among others, attention deficit hyperactivity disorder (ADHD), autism spectrum disorder (ASD), intellectual disability, global developmental delay, developmental language disorder (DLD) as well as motor, learning and communication disorders (3). Although every NDD has distinct features, they share important characteristics: onset in childhood; rather steady course over lifespan; higher prevalence in males; significant overlap with other NDDs and a high heritability although multifactorial in etiology (3, 4).

One of the most common NDD is ADHD, with an estimated prevalence of 3.4% (95% CI: 2.6–4.5) in the general population (5). ADHD is characterized by inappropriate and impairing inattention, motor hyperactivity and impulsivity for the respective developmental level (6). Although much rarer, another prominent NDD is ASD (7). In 2010 the estimated global point prevalence was 1% (8). ASD is characterized by persistent deficits in communication, social interactions and restricted, repetitive patterns of behaviour, interests, or activities (2). Both, ASD and ADHD share high rates of comorbid intellectual disability (9): ADHD was diagnosed in 40%–70% of patients with ASD (10), and vice versa, ASD symptoms were found in 21% of patients with ADHD (11). Additionally, ASD and ADHD are often accompanied by other NDDs, such as intellectual disability (9, 12), language problems (13), global developmental delay (14) and motor disorder (15, 16). Although NDDs are highly heritable and various genes have been associated with them, distinct pathogenetic pathways have yet to be determined (17).

The gene HNF1B is also known as transcription factor 2 (TRF2) and early expression is seen in the kidney, liver, bile ducts, thymus, genital tract, pancreas, lung, and gut (18). Originally, variations in the *hepatocyte nuclear factor-1 β* (HNF-1β, OMIM 189907) have been discovered in patients with maturity-onset diabetes of the young (19), later it has been found to be one major cause of the renal cysts and diabetes syndrome [RCAD (20); OMIM 137920]. Now it is deemed a multi-system disorder, leading to end-stage kidney disease in a subset of patients, including extra-renal symptoms like pancreatic dysplasia, elevated liver enzymes and genital tract abnormalities (21–23).

Around half of the mutations of HNF1B consist of a whole-gene deletion (43%–64%) (24–26) This is caused in almost all cases by a microdeletion on chromosome 17q12, spanning a minimum of 1.4 Mb. In this review, whole gene deletions are therefore labeled 17q12 microdeletions (23, 24, 27). Both, mutations and17q12 microdeletions can arise *de novo*, present in around 50% of the cases (24, 25). In these cases, parents are generally genetically unaffected.

Considering the different kinds of genetic variations in HNF1B (17q12 microdeletions or intragenic mutations), no consistent genotype-phenotype correlation has so far been identified (25). Instead, phenotypes resulting from intragenic HNF1B mutations or 17q12 microdeletions are very heterogenic with a large intra- and interfamilial variability. This makes it likely, that the haploinsufficiency of HNF1B, meaning alterations of gene dosage, leads to the described multi-system disease (22, 28, 29).

From early on, a case report of patients with cognitive impairment emerged (30). Since then, there have been constantly new findings about the phenotype resulting from heterozygous mutations or whole-gene deletions of HNF1B (31–34).

Some conditions seem to be linked to 17q12 microdeletions rather than to intragenic mutations. This is the case for the Mayer-Rokitansky-Küster-Hauser syndrome (35, 36) but also NDDs, including intellectual disability (IQ < 70; ID), global developmental delay and ASD (37, 38). As there have also been reports about NDDs in patients with intragenic mutations (39), NDDs might not be exclusively linked to the 17q12 microdeletion. However, it is unknown whether the risk for NDDs differs significantly in patients with a 17q12 microdeletion from patients with an intragenic HNF1B mutation. Also, little systematical research exists so far to evaluate whether patients with variations in HNF1B have a higher risk for NDDs than the general population.

### Research question

This review aims to answer the following research question: How high is the prevalence of NDDS in patients with 17q12 microdeletions and mutations of HNF1B and exceeds this prevalence the prevalence of NDDs in the general population? To answer this question, we will review findings obtained in original research. We will summarize the existing literature and investigate disease-related characteristics and the observed prevalence of NDDs in patients with a mutation of HNF1B and a 17q12 microdeletion, respectively. Further, we will compare the observed prevalence of NDDs in patients with a mutation of HNF1B and a 17q12 microdeletion with the prevalence of NDDs in the general population in the discussion section.

## Methods

### Literature search

**Figure 1:**
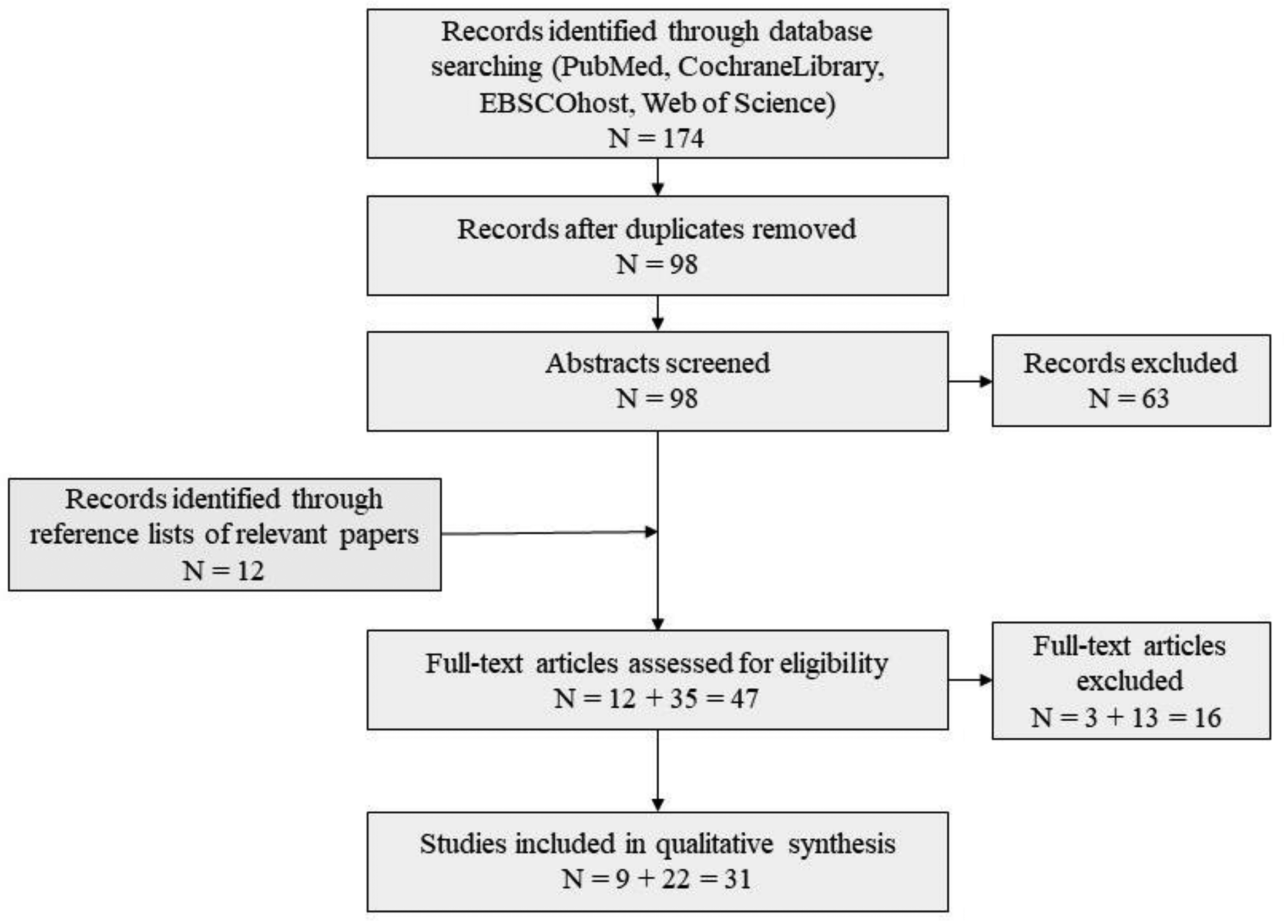
Flow chart of literature search.

A literature research was performed on PubMed, Cochrane library, Web of Science and EBSCO host (selecting the CINAHL, APA PsycInfo, APA PsycArticles and MEDLINE databases), using the keywords autis* OR psychiatr* OR mental OR cognitive OR neurodevelopment* OR neuropsychol* AND HNF1B OR HNF1β OR 17q12 deletion OR 17q12 microdeletion OR TCF-2 OR TCF2 (=Transcription Factor 2, synonym of HNF1B), focusing on the years 1997–2022. The exact search terms were adapted to the requirements of the respective search engine. A total of 174 search results were obtained (see Figure 1). After doublets were removed a total of 98 papers remained. Included were originally published papers in English, which described patients with an HNF1B variations for which also NDD were reported. 22 papers met these criteria, nine additional studies were identified *via* reference lists of relevant papers, therefore a total of 31 papers are reported in this review (see Appendix A). The papers were reviewed by two researchers independently (F. D., C. M. N.) and in case of a contradictory decision, the paper was discussed with a third person (I. K.-B.).

### Descriptive and statistical data analysis

The sample size, the number of cases with NDDs and percentages of NDDs in patients with a HNF1B mutation and 17q12 microdeletion were documented for every paper found (see Table 1). If obtainable, patient characteristics associated with their renal cystic disease were documented separately for patients with a 17q12 microdeletion or HNF1B mutation. To assess group differences for patients with a 17q12 microdeletion or HNF1B mutation regarding their illness-related characteristics, a *χ*^2^ test was calculated. If the expected counts in the contingency table were less than five, Fishers exact test was applied. The reported age in the studies was compared for the two groups using a Mann–Whitney-*U*-Test, as the requirements for a *t*-Test were not fulfilled. For every type of NDD, the observed prevalence was calculated. For that, the number of cases with a certain type of NDD, e.g., ASD was divided by the pooled sample size of studies investigating this type of NDD. Again, to assess group differences for patients with a 17q12 microdeletion or HNF1B mutation regarding the observed prevalence for different NDDs, a *χ*^2^ test was calculated. If the expected counts in the contingency table were less than 5, Fishers exact test was applied (see Table 2). To compare the risk for patients with 17q12 microdeletions and HNF1B mutations regarding NDDs, odd ratios for every study were calculated if the studies provided sufficient data. Odd ratios based on the pooled data for every NDD were calculated as well.

**Table 1:** Percentages of neurodevelopmental disorders (NDD) in studies reporting on NDDs in patients with 17q12 microdeletions and HNF1B mutations.

Source	Focus of paper	*n* total sample (Del/Mut)	NDD Del		NDD Mut		References
			*n*	(%)	*n*	(%)	
Bingham et al., 2001	Case series, genetic etiology in four unrelated families with glomerulocystic kidney disease	5 (-/5)	–	–	1	(20%)	(30)
Shihara et al., 2004	Case study, phenotype of patient with HNF1B mutation	1 (-/1)	–	–	1	(100%)	(40)
Müller et al., 2006	Case study, phenotype of patient with HNF1B deletion	1 (1/-)	1	(100%)	–	–	(41)
Cheroki et al., 2008	Case study, genetic etiology of 14 female patients with Müllerian aplasia, recruited from public health services	1 (1/-)	1	(100%)	–	–	(42)
Raile et al., 2009	995 children and adolescents with diabetes, 50 analyzed for MODY-gene defects, 5 cases of 17q12 microdeletion found. One patient already described in Müller et al, 2006 in greater detail	4 (4/-)	1	(25%)	–	–	(43)
Aggarwal et al., 2010	Case study, patient with RCAD and 17q12 microdeletion	1 (1/-)	1	(100%)	–	–	(44)
Heidet et al., 2010	Retrospective data collection of 377 patients with pathogenic kidney phenotypes, 75 cases with HNF1B deletions/mutations	75 (42/33)	1	(2.4%)	–	(0%)	(25)
Loirat et al., 2010	Report of NDDs in a cohort of 86 children with cystic kidneys and HNF1B molecular anomalies	86 (53/33)	3	(5.7%)	–	(0%)	(33)
Nagamani et al., 2010	Case series, phenotype of patients with micro17q12 microdeletion	4 (4/-)	3	(75%)	–	–	(45)
Faguer et al., 2011	Clinical presentation, imaging findings, genetic changes, and disease progression in adults with HNF1B variations	27 (16/11)	1	(6.3%)	3	(27.3%)	(39)
Moreno-De-Luca et al., 2011	Genetic analysis of 15.749 patients with NDD referred for clinical testing, further clinical and neuropsychological assessment of nine of these patients	9 (9/-)	8	(88.9%)	–	–	(37)
Dixit et al., 2012	Case series, phenotype of three patients with 17q12 microdeletion	3 (3/-)	2	(66.7%)	–	–	(46)
George et al., 2012	Case series, phenotype of three family members with 17q12 microdeletion	3 (3/-)	3	(100%)	–	–	(47)
Palumbo et al., 2014	Case study, phenotype of patient with HNF1B deletion	1 (1/-)	1	(100%)	–	–	(48)
Roberts et al., 2014	Case study, phenotype of patient with HNF1B deletion	1 (1/-)	1	(100%)	–	–	(49)
Jones et al., 2015	Case series, phenotype of two families with HNF1B deletions	5 (5/-)	2	(40%)	–	–	(50)
Laffargue et al., 2015	Comparison of neuropsychological disorders in 27 patients with 17q12 microdeletion and mutations	27 (16/11)	6	(37.5%)	2	(18.2%)	(27)
Clissold et al., 2016	NDDs in 38 patients with HNF1B related diseases	38 (20/18)	8	(40%)	–	(0%)	(31)
Gilboa et al., 2016	Prenatal diagnosis and disease progression in four children with 17q12 deletion	4 (4/-)	4	(100%)	–	–	(51)
Rasmussen et al., 2016	Nationwide register of conducted chromosomal microarray analysis; during 8 yrs 11,216 CMA have been carried out and 12 patients with 17q12 microdeletion have been identified	12 (12/-)	9	(75%)	–	–	(34)
Clissold et al., 2018	DNA methylation profile of HNF1B deletion/mutation groups, sample matched for age, gender, presence of Diabetes; Data retrospectively collected	40 (20/20)	2	(10%)	–	(0%)	(52)
Dubois-Laforgue et al., 2017	ID in an adult cohort with HNF1B-MODY	107 (53/54)	16	(30.2%)	8	14.8%	(53)
Li et al., 2019	Case study, phenotype of patient with HNF1B deletion	1 (1/-)	1	(100%)	–	–	(54)
Nagano et al., 2019	596 patients with renal dysfunctions screened for HNF1B aberrations, which were found in 33 cases (17q12 deletion), further genetic/genotype–phenotype analysis	33 (14/19)	4	(35.7%)	–	(0%)	(38)
Vasileiou et al., 2019	Case series, phenotype of seven patient with 17q12 microdeletion referred for genetic testing	7 (7/-)	5	(71%)	–	–	(55)
Wan et al., 2019	Retrospective data collection of prenatal ultrasound diagnosis in 2,161 pregnancies in 2 yrs. 126 cases of renal abnormalities and 5 cases with 17q12 microdeletions, 2 pregnancies were terminated	3 (3/-)	1	(33.3%)	–	–	(56)
Lalieve et al., 2020	School level of 166 children was analysed from a total of 223 eligible patients with HNF1B deletion/mutation	166 (110/56)	14	(12.7%)	2	(3.6%)	(32)
Lim et al., 2020	Genotype and phenotype unrelated paediatric patients recruited *via* genetic testing	14 (6/8)	1	(16.7%)	2	(25%)	(57)
Bulu et al, 2021	Case study, patient with microdeletion of 17q11.2-17q12 chromosome region and bipolar disorder I	1 (1/-)	1	(100%)	–	–	(58)
Ng et al., 2021	Phenotype of patients with HNF1B-MODY identified from MODY screening study	12 (2/10)	1	(50%)	–	(0%)	(59)
Wu et al., 2021	Case series, phenotype of four family members with HNF1B deletion	4 (4/-)	2	(50%)	–	–	(60)
	**Overall (Mean %)**	**695** (**279/416)**	**105**	(**25%)**	**19**	(**6.8%)**	

The following abbreviations were used NDD, neurodevelopmental disorder; Del, 17q12 microdeletion; Mut, mutation of HNF1B; MODY, maturity onset diabetes of the young; RCAD, renal cyst and diabetes syndrom.

**Table 2:** Types of NDDs in patients with a 17q12 microdeletion or mutation of HNF1B.

	17q12 microdeletion		Mutation				Odd Ratio (Deletion/Mutation)
	Observed prevalence, n/N investigated	(%)	Observed prevalence, n/N investigated	(%)	*χ*^2^ (1)	*p*	
Any NDD	**105**/**416**	(25.2%)	**19/279**	(6.8%)	**309**	.001*	0.4
ID	**28**/**183**	(15.3%)	**9/73**	(12.3%)	**0.37**	.542	1.7
LD	**61**/**245**	(24.5%)	**12/127**	(9.5%)	**12.65**	.001*	0.6
GDD	**6**/**34**	(17.7%)	**–**		**–^a^**	**–**	
DLD	**28**/**107**	(26.2%)	**1/8**	(12.5%)	**0.74^b^**	.677	3.4
MD	**29**/**116**	(24.7%)	**–**		**–**	**–**	
ASD	**22**/**158**	(13.3%)	**–**		**–**	**–**	
Autistic features^b^	**7**/**22**	(31.8%)	**–**		**–**	–	
ADHD	**7**/**29**	(24.1%)	**1/8**	(12.5%)	**0.50^b^**	.655	4.3
Epilepsy	**5**/**23**	(26.1%)	**3/12**	(16.7%)	**0.48^b^**	1	3.4

*χ*^2^ statistic with exact significance (two sided).

^a^ No statistic was calculated because at least one variable in the 2 × 2 contingency table is a constant.

^b^ Assumption that no expected frequency is below five was not met, so Fishers exact test 2 × 2 contingency tables (two sided) was calculated. ID, intellectual disability with IQ < 70; GDD, global developmental delay; DLD, delayed language development; MD, motor disorder; ASD, autism spectrum disorder.

^c^ Autistic features are not a formal diagnosis of NDD but were reported because of the emphasis on ASD in the present review. ADHD, attention deficit hyperactive disorder.

* *p* < 0.001, two-sided.

## Results

We will first qualitatively summarize the existing literature, then we will present the data analysis of the studies. We will summarize the 31 studies identified on NDDs in patients with variations in HNF1B and will divide this section in single case studies/case series and larger samples of patients.

### Single case studies and case series

Several studies reported on patients with HNF1B variations in single case studies or case series. Considerably more case studies on patients with a 17q12 microdeletion (41, 42, 44–46, 48–50, 54, 58, 60) have been published than on patients with a mutation of HNF1B (40).

In the single case studies, all patients with a 17q12 microdeletion were reported to have some kind of NDD, including intellectual disability (41, 44, 48), ADHD (48, 58), or ASD (49). The patient in the single case study with a mutation of HNF1B was reported to have a learning disorder (40).

The case series presented either findings from sets of family members (30, 50, 60) or unrelated patients (45, 46, 55) referred for genetic testing. In the studies reporting on NDDs in family members, the observed prevalence of NDDs ranged from 20% (30) in patients with a mutation of HNF1B to 40% (50), and 100% respectively (47) in patients with a 17q12 microdeletion. In patients with a 17q12 microdeletion, cases of learning disorder, ASD, global developmental delay and motor disorder were observed (47, 50, 60), in patients with a mutation of HNF1B, a case of learning disorder was observed (30). In studies reporting on NDDs in unrelated patients, the observed prevalence of NDDs ranged from 66.7% (46) to 75% (45) in patients with a 17q12 microdeletion. In patients with a 17q12 microdeletion, cases of ASD, intellectual disability, learning disorder, developmental language disorder, motor disorder and epilepsy were observed (45, 46, 55). None of the case series in unrelated patients reported on NDDs in patients with a mutation of HNF1B.

### Studies with larger sample sizes

Three studies tested for variations in HNF1B in larger samples of patients with pathogenic kidney phenotypes (25, 38) and diabetes (43). In these samples, patients with a 17q12 microdeletion mostly presented with intellectual disability, with a prevalence ranging from 2.4% to 25% (25, 38, 43). In patients with a mutation of HNF1B, no NDD was found. Two study tested for variations in HNF1B in a large sample of patients referred to genetic testing because of a NDD (34, 37). All patients had a 17q12 microdeletion and presented mostly with developmental language disorder (0.6% (34)–67% (37)), motor disorder (33% (34)–78% (37)) and learning disorder (11% (37)–58% (34)). The reported prevalence for ASD ranged from 8% (34) to 44% (37)).

Two studies followed up cases with 17q12 microdeletions which were detected prenatally (51, 56). While one study describes just one case with motor disorder and comorbid developmental language disorder (56), the other study describes NDDs in all four patients, three with ASD and one with motor disorder (51).

Two studies concentrated on the intellectual abilities of patients with variations in HNF1B (32, 53). In patients with a 17q12 microdeletion, they found a prevalence of 17% (53) for intellectual disability, the prevalence for learning disorders ranged from 13% (32) to 26% (53). In patients with a mutation of HNF1B, the prevalence for intellectual disability was 11% (53), the reported prevalence for learning disorder ranged from 4% (32) to 11% (53).

Three studies specifically focused on the occurrence of NDDs in patients with HNF1B variations and differences between patients with deletions and mutations in HNF1B (27, 31, 33). Patients with a 17q12 microdeletion presented mostly with learning disorder (23% (27)—30% (31)) and ASD (4% (27)–20% (31)). Only one study found NDDs in patients with a mutation of HNF1B (27), two patients were reported to have learning disorder (15%).

Four studies targeted somatic features of the disease and report NDDs as a secondary aspect (39, 52, 57, 59.) In patients with a 17q12 microdeletion the following NDDs were found: intellectual disability [6% (39)], ASD [5% (52)], global developmental delay (5% (52)–50% (59)), ADHD [17% (57)] and ASD [5% (52)]. Two studies reported on NDDs in patients with a mutation of HNF1B (39, 57), here, patients presented mostly with intellectual disability (13% (57)–18% (39)).

### Descriptive and statistical data analysis

A total of 31 studies was identified, comprising 695 patients with variations in the HNF1B gene, among these 416 patients with a 17q12 microdeletion and 279 patients with a mutation of HNF1B. The focus of each paper and the observed prevalence of NDDs in each HNF1B deletion/mutation sample is summarized in Table 1. Of the 416 patients with a deletion, 105 (25%) were reported to have any NDD. Of the 279 patients with a mutation, 19 (7%) had any NDD.

A total of 97 case descriptions of HNF1B-related kidney disease in combination with a NDD on an individual level were available in 29 papers. The remaining two papers did not offer information on an individual level (27, 32). The characteristics and clinical phenotypes of patients with deletions or mutations, respectively, are reported in Table 2. Patients with a 17q12 microdeletion differed from patients with a mutation regarding age (*p* = .004), kidney abnormalities (*p* = .018) and diabetes (*p* = .015). Patients with a mutation were older and suffered from kidney abnormalities/failures and diabetes more frequently.

The observed prevalence for different type of NDDs was calculated for patients with a 17q12 microdeletion or mutation separately (see Table 3). Patients with a 17q12 microdeletion differed from patients with a mutation regarding the observed prevalence of any NDD (*p* = .001) and learning disorder (*p* = .001). Compared to patients with a mutation, patients with a 17q12 microdeletion had a higher prevalence for any NDD and specifically learning disorder. We were not able to compare the observed prevalence of ADHD and ASD because of small sample sizes.

**Table 3:** Characteristics of patients with NDDs.

	17q12 microdeletion (*n* = 82)		Mutation (*n* = 14)			
	*N*	(%)	*N*	%	*χ*^2^ (1)	*p*
Mean age (SD)^a^	**22.68** (**16.81)**		**39.73** (**23.19)**			sig.
Sex	** **		** **		**1**.**90**	0.169
Male	**42**	(51%)	**9**	(64%)	** **	
Female	**36**	(44%)	**3**	(21%)	** **	
Inheritance	** **		** **		** **	
De novo	**26**	(32%)	**1**	(7%)	**1**.**52^b^**	.265
Inherited	**12**	(15%)	**2**	(14%)	** **	
Renal abnormalities/failure	**58**	(71%)	**14**	(100%)	**5**.**46^b^**	.018*
Diabetes	**30**	(37%)	**10**	(71%)	**5**.**97**	.015*
Extra-renal features	**37**	(45%)	**10**	(71%)	**3**.**31**	.069
Comorbid psychiatric disorders	**9**	(11%)	**0**	(0%)	–^c^	–

Percentages are calculated for the 17q12 microdeletion and mutation group separately. Percentages might not add up to 100% in some cases, as information was not available for every patient.

^a^ To calculate for age differences between the groups, a Mann–Whitney-*U*-Test was conducted, as the normal distribution was not given. Median Age for the 17q12 microdeletion group was 40. 6 and for the Mutation group was 63.5, with *U* = 192.5, *Z* = −2.846, *p* = .004.

^b^ Assumption that no expected frequency is below five was not met, so Fishers exact test 2 × 2 contingency tables (two sided) was calculated.

^c^ No statistic was calculated because at least one variable in the 2 × 2 contingency table is a constant.

* *p* < 0.05, two-sided.

If sufficient data was presented in an original study, odd rations were computed. Despite of the descriptive differences, none of the studies showed a significant difference between patients with a 17q12 microdeletion and a mutation of HNF1B with regard to NDDs (OR = 5.6, 95% CI [0.5 63.28] (39); OR = 0.8, 95% CI [0.1 4.6] (27); OR = 0.1, 95% CI [0.03 0.2] (53); OR = 0.3, 95% CI [0.1 1.2] (32); OR = 1.7, 95% CI [0.1 24.3]).

### Comparing the prevalence of NDDs in patients with HNF1B variations to the prevalence of NDDs in the general population

A recent study shows that up to 17.3% of children and adolescents between 3 and 17 years in the United States of America are affected by NDDs (61). Accordingly, the observed prevalence of NDDs is slightly higher in patients with deletions of HNF1B and lower in patience with mutations than in the general population. As stated by the DSM-5 (2), the prevalence for intellectual disability is 1%, for learning disorder 5%–15% in school children and 4% in adults, for motor disorder 5%–6% in children (5–11 years), for ASD 1%, ADHD 5% in children and 2.5% in adults. The prevalence for global developmental delay ranges from 1% to 3% in children (62), the prevalence for developmental language disorder is 7.5% (63) and for epilepsy 0.76% (64). So far, the observed prevalence of the above named NDDs in patients with deletions of HNF1B (25.2%) as well as mutations of HNF1B (6.8%) appears to exceed the reported prevalence in the general population.

## Discussion

### Summary of findings

This review had the purpose to summarize and compare the NDDs that have been reported for patients with a 17q12 microdeletion or intragenic mutation of HNF1B regarding the frequency and type of NDDs. The analysis of the 31 studies showed that NDDs have so far been reported considerably more often in patients with a 17q12 microdeletion compared to patients with an intragenic mutation of HNF1B (25% vs. 7%). Statistically, patients with deletions were more likely to present with NDD as well as learning disorder than patients with mutations. In contrast, patients with mutations presented with diabetes and kidney abnormalities/failure more frequently which seemed to be related to their higher age.

Patients with a 17q12 microdeletion seem to suffer from a wider variety of NDDs than patients with a mutation: patients with a 17q12 microdeletion presented with intellectual disability, learning disorder, global developmental delay, developmental language disorder, motor disorder; ASD, ADHD, epilepsy and/or autistic features (although formally not a NDD), while patients with a mutation presented only with intellectual disability, learning disorder, developmental language disorder, ADHD and/or epilepsy.

### Mechanisms linking NDDs to HNF1B variations

So far, no mechanism has been determined that leads to the differences in the phenotype regarding NDDs of the two carrier groups of HNF1B alterations. A possible influence of HNF1B on the development of the hindbrain has been observed in mice and zebrafish models (65, 66), but not in humans yet. As NDDs have also been found in patients with a HNF1B mutation, the haploinsufficiency as one risk factor for NDDs can still be regarded as a possible cause. Nevertheless, other genes in the deleted region have also been suggested to play a role in the NDDs that can be observed in patients with 17q12 microdeletions. These include the gene LHX1 (37, 48, 60), which has been shown to be crucial for the migration of interneurons originating in the pre-optic area during embryonic cortical development (67). Two other genes, PIGW and PCGF2, are also hypothesized to be candidates for NDDs (55, 60) as genetic variations of those two genes have been linked with developmental delay and/or epilepsy (68–70) In addition, Laffargue et al. (27) suggested that an interaction between HNF1B and other transcription factors like homeobox protein Hox-A1 could cause NDD, as an interaction of those two transcription factors has been found during mural hindbrain development (71). Additional or synergistic effects due to “second-site” CNVs on top of a primary genomic rearrangement have been found for other genomic disorders (e.g., 16p11.2 duplication) and are also possible to play are role for 17q12 microdeletions. In a large cohort of children with syndromic ID and congenital abnormalities, 10% of the children carried a secondary CNVs (72). These effects could influence neurodevelopmental pathways and the disease outcome, e.g., the phenotype and severity of the disease, contributing to the additional NDDs.

### Validity of estimations

Percentages of reported frequency of NDD in patients with HNF1B gene variations (i.e., 2.4%–100%) vary greatly; i.e., for patients with a mutation from 0% to 18.2% and for patients with a 17q12microdeletion from 2.4% to 100%. This might be due to the characteristics of the particular study. For instance, percentages tended to be higher, the smaller the samples were (e.g., *n* = 4, 100% in (51) or *n *= 5, 40% in (43)) or the more detailed the patients were described (34); *n* = 12; 75%). Papers who did not focus on psychological aspects tended to report a lower percentage of NDDs [e.g., (25); *n* = 42 patients with a 17q12microdeletion, 2.4%] as well as papers with bigger samples and reduced psychological assessment [e.g., (32); *n* = 110 with a deletion; 14%]. Overall, this points to the existence of a publication bias and limits the validity of the prevalence estimations.

When comparing the prevalence of NDDs in patients with HNF1B variations to the prevalence of NDDs in the general population, it strikes as odd that the prevalence of NDDs in patients with a mutation of HNF1B should be lower than in the general population. A reason for this low prevalence estimation of NDDs in patients with mutation might be a sampling bias. And the true prevalence might be higher.

The validity of the prevalence estimations are also limited by insufficient information regarding the diagnostic of NDDs in the reviewed studies. Only in three studies all participants underwent a standardized intelligence test (44, 47, 49), in six studies either only subgroups were assessed or information about the assessment was missing, e.g., name of the test (27, 37, 53–55, 58). Of 11 studies reporting on ASD cases, only three studies described the use of any diagnostic tool (27, 33, 37) and only for one patient with ASD (37) the diagnosis was assigned according to the NICE guidelines (73). Although comorbid NDDs or mental disorders were often reported, information whether comorbidities were routinely assessed were missing in the reviewed studies. However, the assessment of comorbid disorders is central for the diagnosis of ASD (73).

Further, the studies did not report the use of appropriate assessment tools for any NDDs, like standardized interviews (74, 75). Instead, information on the diagnostic process were missing or special education needs as indicators for NDDs, especially learning disorder were used (32, 34). Although, special education needs point to the existence of a NDD, they are not a valid diagnostic tool.

Overall, sample sizes were small. Only seven studies had a sample size above 30 patients (see Table 1) and only four studies investigated group differences based on inferential statistics (27, 31, 32, 53).

Overall, the small sample sizes and the quality of studies, especially regarding the diagnosis of NDDs make it impossible to draw valid conclusions about prevalence ratings in samples with HNF1B variations just yet.

## Limitations

One large obstacle is the sparsity of HNF1B-associated disorders. Population based studies estimate a prevalence for 17q12 microdeletion of approximately 0.023%–0.025% (76, 77). In samples of patients with kidney abnormalities, HNF1B alterations can be found in about 5%–31% depending on the specific sample characteristics (22). The number of patients who present with an additional NDD is therefore even more limited. This reflects in the lack of good-quality studies with large, matched samples regarding age, sex and factors regarding kidney disease and diabetes.

As for a long time HNF1B-related kidney disease was regarded as primarily a physical disease, some papers report NDDs only as a “side note”. This might influence this review in several ways: In larger samples, NDDs as well as mild psychological symptoms or impairments might have easily been overlooked leading to possibly underestimated numbers. On the other hand, a publication bias might have led to an overestimation of NDDs in patients with HNF1B alterations.

Lastly, regarding the comparison of studied that focused on NDDs in particular, differences in the sensitivity of criteria for NDD (e.g., neuropsychological assessments vs. special education needs; learning disability vs. learning difficulties) and therefore differences in the percentages of NDD make it hard to compare these studies (27, 31, 32).

## Conclusion

This review shows that NDDs are frequently found in patients with HNF1B variations. It seems that they are more common in patients with 17q12 microdeletion than in patients with mutation of HNF1B. The observed prevalence for NDDs, including ASD was found to be higher patients with 17q12 microdeletion than in the general population but the validity of the prevalence estimates are limited by the insufficient quality of the studies.

A first step to enhance data quality includes considering NDDs as a further possible symptom of patients with a deletion. This should include consistent reporting of existence or absence of NDDs in scientific papers as well as considering possible impairments during the clinical routine. Further studies will be needed to evaluate the neuropsychological profiles of patients with a 17q12 microdeletion or mutation of HNF1B, including matched samples and a significant sample size for statistical comparison.

If a standardized assessment of symptoms of NDD would be integrated in the clinical routine of patients with HNF1B variations, especially in patients with deletions of HNF1B, access to valuable treatment options could be facilitated and subsequently, the quality of life of patients and their families could be enhanced.

The authors are part of the NEOCYST consortium (www.neocyst.de/en/), a multicenter, interdisciplinary network of clinicians and scientists exploring early onset cystic kidney diseases and undertake a research project to assess neuropsychological symptoms in patients with HNF1B variations and other cystic kidney diseases.

## Appendix A

A literature research was performed on PubMed, Cochrane library, Web of Science and EBSCO host (selecting the CINAHL, APA PsycInfo, APA PsycArticles and MEDLINE databases), using the keywords autis* OR psychiatr* OR mental OR cognitive OR neurodevelopment* OR neuropsychol* AND HNF1B OR HNF1β OR 17q12 deletion OR 17q12 microdeletion OR TCF-2 OR TCF2 (= Transcription Factor 2, synonym of HNF1B), focusing on the years 1997-2022. The exact search terms were adapted to the requirements of the respective search engine. A total of 174 search results were obtained. After doublets were removed a total of 98 papers remained. Included were originally published papers in English, which described patients with an HNF1B variations for which also NDD were reported. 22 papers met these criteria, nine additional studies were identified via reference lists of relevant papers therefore a total of 31 papers are reported in this review (see Figure 1). The papers were reviewed by two research independently (F. D., C. M. N.) and in case of a contradictory decision, the paper was discussed with a third person (I. K.-B.).
